# The use of auditory and visual context in speech perception by listeners with normal hearing and listeners with cochlear implants

**DOI:** 10.3389/fpsyg.2013.00824

**Published:** 2013-11-05

**Authors:** Matthew B. Winn, Ariane E. Rhone, Monita Chatterjee, William J. Idsardi

**Affiliations:** ^1^Waisman Center & Department of Surgery, University of Wisconsin-Madison, Madison, WI, USA; ^2^Department of Neurosurgery, University of Iowa, Iowa City, IA, USA; ^3^Boys Town National Research Hospital, Omaha, NE, USA; ^4^Department of Linguistics, University of Maryland College Park, College Park, MD, USA

**Keywords:** cochlear implants, speech perception, context effects, spectral degradation, multisensory, audio-visual integration

## Abstract

There is a wide range of acoustic and visual variability across different talkers and different speaking contexts. Listeners with normal hearing (NH) accommodate that variability in ways that facilitate efficient perception, but it is not known whether listeners with cochlear implants (CIs) can do the same. In this study, listeners with NH and listeners with CIs were tested for accommodation to auditory and visual phonetic contexts created by gender-driven speech differences as well as vowel coarticulation and lip rounding in both consonants and vowels. Accommodation was measured as the shifting of perceptual boundaries between /s/ and /∫/ sounds in various contexts, as modeled by mixed-effects logistic regression. Owing to the spectral contrasts thought to underlie these context effects, CI listeners were predicted to perform poorly, but showed considerable success. Listeners with CIs not only showed sensitivity to auditory cues to gender, they were also able to use visual cues to gender (i.e., faces) as a supplement or proxy for information in the acoustic domain, in a pattern that was not observed for listeners with NH. Spectrally-degraded stimuli heard by listeners with NH generally did not elicit strong context effects, underscoring the limitations of noise vocoders and/or the importance of experience with electric hearing. Visual cues for consonant lip rounding and vowel lip rounding were perceived in a manner consistent with coarticulation and were generally used more heavily by listeners with CIs. Results suggest that listeners with CIs are able to accommodate various sources of acoustic variability either by attending to appropriate acoustic cues or by inferring them via the visual signal.

## Introduction

Variability in the acoustic realization of speech segments is a well-known phenomenon that can arise from several sources, including coarticulation with neighboring segments and inter-talker differences related to gender and vocal tract size. Despite this variability in the physical properties of the signal, normal hearing (NH) listeners are remarkably successful at perceiving and understanding speech. Listeners are thought to accommodate this variability by compensating for the context in which sounds are heard and thus, recognize that two different sounds are really the “same.” In this paper, we explore this phenomenon for listeners with cochlear implants (CIs) and for listeners with NH, in normal (unprocessed) or CI-simulated conditions.

The sounds heard by CI listeners are spectro-temporally degraded and altered by the device because of the limited number of independent spectral processing channels (Fishman et al., 1997; Friesen et al., 2001) and electrical interaction between the electrodes that carry information from those channels (Chatterjee and Shannon, 1998; Abbas et al., 2004; Hughes and Stille, 2009). The coding of sounds throughout their auditory pathways is likely to be additionally impaired relative to their NH peers, and CI listeners also experience a distorted tonotopic representation of sound (Fu and Shannon, 1999), owing to limitations in surgical placement of the electrode array along the basilar membrane. As a result of these factors, listeners with CIs have much poorer spectral resolution than listeners with NH (Loizou and Poroy, 2001; Henry et al., 2005; Won et al., 2007). Acoustic simulations of CI processing confirm, however, that NH listeners can understand speech with a high degree of accuracy even when signals are spectrally degraded (Shannon et al., 1995; Friesen et al., 2001). It is likely that in these simulations, listeners capitalize on the redundancy of acoustic information in the speech signal to compensate for spectral information that is lost, to ultimately reach an adequate level of performance. Analyses of the contribution of spectral and temporal cues validate this hypothesis; phoneme identification performance deficits resulting from decreased spectral resolution can be counteracted by increased temporal resolution, and vice-versa (Xu et al., 2005). Furthermore, perceptual weighting for phonetic cues decreases in the spectral domain and increases in the temporal domain for listeners with CIs or for NH listeners in CI simulations (Winn et al., 2012). Thus, bottom-up and top-down processes associated with context-dependent speech perception may be different for CI listeners. It is our view that the evaluation of listeners with CIs should include not only their overall performance on identifying speech sounds, but also whether their auditory systems support the kind of contextual accommodation that is necessary for real-world communication (e.g., with multiple conversation partners and in a variety of phonetic environments). However, it remains unknown to what extent listeners with CIs can accommodate contextual variability in phonetic identification.

For NH listeners, accommodation to phonetic context is observed across many different types of target sounds and in various contexts. A heavily explored example is the perception of stop consonants /g/ and /d/ in the context of liquid consonants /l/ or /r/. Given an ambiguous syllable that sounds like either /da/ or /ga/, listeners are biased to hear /da/ if a preceding syllable is /ar/, but biased to hear /ga/ if the preceding syllable is /al/ (Mann, 1980). This effect persists even if the precursor syllable is a different voice or a non-speech tone sweep (Lotto and Kluender, 1998), and is also observed in native speakers of Japanese, for whom the /l/ and /r/ precursors are not phonologically contrastive (Mann, 1986). The general trend that emerges from these investigations is that sounds with higher-frequency components (e.g., /d/) are facilitated by precursor sounds with lower-frequency energy (e.g., /r/), and vice-versa. The high-low contrast enhancement neatly frames the /rd/-/lg/ effect and has been observed for other speech sound sequences as well For example, sounds that are ambiguous between /t/ and /k/ are heard more often as /t/ (high) after /∫/ (low) sounds but heard as /k/(low) after /s/ (high) sounds (Mann and Repp, 1981). Although frequency/spectral contrast by itself is not universally accepted as the best explanation for contextually dependent perception (Fowler, 2006; Viswanathan et al., 2009), we expect that the auditory perception of other phonetic primitives (e.g., gestures) should also depend at least partly on spectral resolution in the auditory system.

The importance of sound frequency contrast should be problematic for CI listeners on the basis of their impaired auditory systems and the poor frequency coding of CIs. Various studies have shown, however, that listeners can use non-auditory information such as visual information and other, indirect, information about the speaker and the speech signal when accommodating phonetic variability. For example, listeners can incorporate lexical knowledge into phonetic perception; Elman and McClelland, 1988 found that fricative perception was affected by whether a precursor was a real word or non-word. Strand and Johnson (1996) showed that listeners can use visual cues for talker gender to accommodate gender-related phonetic differences (a finding that will be further explored in this paper). Fowler et al. (2000) showed that visual cues to a precursor syllable can influence perception of a subsequent syllable in a manner similar to that shown with auditory stimuli by Lotto and Kluender (1998). Although Holt et al. (2005) critique various accounts of visually-mediated effects as *context per se*, the evidence for auditory-visual integration in speech perception is well established, with well-known examples such as Sumby and Pollack's (1954) investigation of visual contributions to intelligibility, and the perceptual fusion revealed by the McGurk effect (McGurk and MacDonald, 1976). All of these examples suggest that limitations in the auditory domain can be mitigated by activation of other modalities or cognitive mechanisms.

While the exact mechanisms of accommodation to phonetic context across modalities have yet to be completely elucidated, it is increasingly clear that listeners exploit any information that is available in the signal, under the right circumstances. This behavior appears suited to accommodate coarticulation and other sources of variability in speech production. The extent to which listeners with CIs overcome auditory limitations to accommodate speech variability is largely unknown. In this study, we examine context effects in the auditory and visual domains for listeners with CIs; we compare this with performance of listeners with NH using unprocessed as well as spectrally-degraded speech tokens.

The consonants /∫/ (as in “she”) and /s/ (as in “see”) are the primary focus of our investigation, not for their importance in word identification, but for their well-known patterns of variability across talkers and across different phonetic environments. These sounds are voiceless fricatives that contrast primarily in the amplitude spectrum; spectral peak frequency is higher for /s/ than that for /∫/, and is considered to be a dominant cue in the perceptual distinction (Evers et al., 1998; Jongman et al., 2000; McMurray and Jongman, 2011). Fricatives produced by females have higher-frequency spectral energy than those produced by males (Jongman et al., 2000), and listeners accordingly judge the boundary between /∫/ and /s/ to be at a higher frequency for female than for male voices (Mann and Repp, 1980). Although this is commonly attributed to differences in vocal tract size (Schwartz, 1968), this pattern is at least partly learned. For example, the sex of a child is reliably identifiable via voice prior to pubescent physical changes (Perry et al., 2001), body size shows a weak relationship to vocal tract resonance in adults (González, 2004), and gender-related accommodation to variation in adults' speech is consistent with common societal impressions of sexual orientation (Munson et al., 2006). This is unsurprising in view of various other culturally-specified characteristics of speech. For example, pitch disparity in the speech of male and female voices is greater in Japanese than in Dutch (van Bezooijen, 1995; van Dommelen and Moxness, 1995).

Other factors that affect the production and perception of /∫/ and /s/ sounds include the vowel context in which they are spoken and the formant transitions connecting the fricative to the vowel segment. In the context of a following vowel produced with lip rounding, both of these fricatives show a global frequency-lowering effect, and listeners compensate by accordingly lowering the frequency boundary between them (Kunisaki and Fujisaki, 1977; Mann and Repp, 1980). Additionally, the configuration of formants at the consonant-vowel boundary is affected by the fricative such that the pattern is slightly different for /s/ than for /∫/. Although such formant transitions are not very compelling cues for adult listeners, phoneme identification tends to be quicker when formant transitions in adjacent vowels are consistent with the presented fricative (Whalen, 1984). The influence of context in fricative identification has thus been strongly established and is now an important component of modern theories of speech perception. McMurray and Jongman (2011) modeled fricative categorization with a large number of acoustic cues obtained from a corpus of English fricative-vowel syllables and found best performance by a model that retained talker information (e.g., gender) and vowel context, in addition to certain attributes within the fricative itself. Given the nature of consonant variability across talkers and across vowel environments, listeners should achieve greater success identifying consonants if they are able to incorporate contextual cues from the speaker and from segments adjacent to those consonants.

Few studies have examined the use of context in phonetic perception by CI listeners; results generally suggest limited success in this area. Hedrick and Carney (1997) tested four CI listeners who all demonstrated virtually no use of context in fricative identification; one listener made use of contextual cues in neighboring sounds only when the target sound was designed to be ambiguous. Summerfield et al. (2002) tested 26 adult CI listeners, out of whom 12 made use of vocalic context. In that study however, different kinds of context were not systematically varied; the two contexts tested differed by talker gender, vowel and by formant transitions. Specifically, one context consisted of a male voice speaking /u/, which contained formant transitions that indicated /s/; all three cues should yield bias toward /s/. The other context consisted of a female voice speaking /i/, with formant transitions from /∫/; all of these contexts should yield bias toward /∫/. It remains unclear whether CI listeners can make use of any of those types of context individually, or whether it is necessary to have multiple cues in order to affect phonetic perception. Additionally, of the pediatric CI listeners tested by Summerfield et al., none with less than 2.5 years of implant experience demonstrated any use of vocalic context, in contrast to behavior observed in young children with NH, who demonstrate considerable use of vocalic context in fricative identification (Nittrouer, 1992). On the basis of these results, the use of contextual (vocalic) information by CI listeners appears to be limited, and CI users probably require at least some experience with their devices and perhaps the confluence of multiple cooperating cues in order to facilitate contextual effects in phonetic identification.

Visual cues play a role in accommodating inter-talker differences, and this has implications for listeners with impaired auditory systems. When listening to a gender-atypical talker, listeners with NH shift their perceptual phonetic boundaries based on visual cues to talker gender (Strand and Johnson, 1996; Johnson et al., 1999). For listeners with CIs who have normal vision, visual cues to talker gender should be unimpaired, and the current study explores whether CI listeners can exploit the learned knowledge of gender-related differences via visual cues to accommodate the difference between male and female voices.

In this study, we investigate whether visual cues can aid the accommodation to phonetic context and gender information with listeners who wear CIs, in view of their specific limitations in the auditory domain. We follow a design that is similar to that used by Strand and Johnson (1996), in which listeners identified speech sounds spoken by talkers whose voices did not give clear cues to gender. For listeners with CIs, auditory cues to gender are also unclear, albeit because of auditory and engineering limitations. CI listeners may recruit information in the visual domain to influence phonetic perception. In contrast with Strand and Johnson (1996), we used talkers whose voices are gender-stereotypical, to represent a more standard listening situation. We also test NH listeners using both unprocessed and spectrally-degraded speech to see whether patterns of context effects differ as a function of experience with the degraded auditory input.

In the first experiment, we tested accommodation to phonetic context in the acoustic domain only. Specifically, we sought to clarify the separate effects of formant transitions, vowel context, and auditory gender cues. To examine the effects of spectral degradation separately from the additional factors involved with using a cochlear implant, normal-hearing listeners were also tested with noise vocoded speech (which is regarded by some as a “CI simulation”).

## Experiment 1 (auditory phonetic context effects)

### Materials and methods

#### Participants

Participants included 10 adult (mean age 21.9 years; 8 female) listeners with NH, defined as having pure-tone thresholds ≤20 dB HL (hearing level) from 250 to 8000 Hz in both ears [American National Standards Institute (ANSI), 2007]. A second group of participants included 7 adult (age 50–73; mean age 63.7 years; 3 female) post-lingually deafened cochlear implant users. All were considerably older than our sample of listeners with NH. Six were users of the Cochlear Freedom or N24 devices; one used the Med-El device. See Table 1 for demographic information and speech processor parameters for each CI user. All participants were native speakers of American English and gave informed consent; procedures were approved by the University of Maryland Institutional Review Board.

**Table 1:** **Demographic information about the CI participants**.

**ID No**	**Sex**	**Duration of HL**	**Age at test**	**Years w/CI**	**Device**	**Consonants %**	**Vowels %**	**HINT sentences %**
C12	F	Unknown	66	3	Freedom	66	51	87
C18	F	10 years	66	3	Freedom	68	84	99
C20	M	22 years	64	7	N 24	68	46	93
C25	M	11 years	50	10	N 24	72	69	DNT
C30	M	Unknown	56	2	Med-El	62	29	DNT
C36	F	59 years	71	5	Freedom	83	62	99
C42	F	4 years	73	4	Freedom	70	58	79

DNT: Did not test. % indicates percent correct for closed set vowels, closed set consonants and open-set sentences from the Hearing in Noise Test (Nilsson et al., 1994), presented in quiet.

#### Stimuli

Stimuli consisted of 4 varying parameters: fricative spectrum (9 levels), taker (4 levels; 2 female and 2 male), vowel (2 levels), and formant transitions at vowel onset (2 levels). There were thus a total of 144 (9*4*2*2) stimuli.

***Fricative synthesis***. The fricative components of the stimuli used in this study consisted of a nine-step continuum whose endpoints were modeled after productions of /∫/ and /s/ sounds in real words (“see, she, sue, shoe”) produced by four (two female, two male) native speakers of English at the University of Maryland. Items in the continuum were comprised of three spectral peaks that varied in three covarying dimensions: frequency (center of the noise band), bandwidth, and relative amplitude of the peaks (spectral tilt). In other words, continuum steps gradually morphed from /∫/ to /s/ by three related dimensions. Spectral peak frequencies and bandwidths were interpolated along a log-frequency scale between observed values for /∫/ and /s/. Table 2 contains details of all the parameters of this continuum and fricatives were created using the Praat^1^ software (Boersma and Weenink, 2011) by filtering 180 ms of white noise into peaks of appropriate frequency, bandwidth and amplitude, adding them, and then imposing a uniform amplitude contour featuring 115 ms of rise-time and 18 ms of fall-time, which were representative of these consonants across the recordings that were collected.

**Table 2:** **Acoustic description of the fricative continuum**.

**Continuum step:**	**/∫/ 1**	**2**	**3**	**4**	**5**	**6**	**7**	**8**	**9 /s/**
**PEAK FREQUENCIES (Hz)**									
SP1	2932	3226	3550	3906	4298	4729	5203	5726	6300
SP2	6130	6357	6592	6837	7090	7352	7625	7907	8200
SP3	8100	8283	8472	8666	8863	9065	9272	9484	9700
**BANDWIDTHS (Hz)**									
SP1	1500	1556	1612	1671	1732	1796	1861	1929	2000
SP2	3500	3500	3500	3500	3500	3500	3500	3500	3500
SP3	2520	2670	2828	2997	3175	3364	3564	3775	4000
**AMPLITUDE RELATIVE TO PEAK 2 (dB)**									
SP1	1.67	0.83	0.00	−0.83	−1.67	−2.50	−3.33	−4.17	−5
SP3	−1.7	−0.8	0.0	0.8	1.7	2.5	3.3	4.2	5

The peak frequency continuum steps were interpolated using a log scale ranging from 3.467 to 3.799. Values are presented here in Hz for ease of interpretation. SP1, SP2, SP3 refer to the three spectral peaks from lowest to highest. Bandwidth refers to the bandwidths of spectral peaks 6 dB below the peak level.

***Contexts***. Each step of the fricative continuum was prepended to (and effectively cross-spliced with) one of sixteen vocalic contexts consisting of the /i/ and /u/ vowels from natural recordings from the words “see,” “she,” “sue,” and “shoe” spoken by four native speakers of English (two female and two male, all phonetically trained, one of whom was the first author). The /i/ vowels from “see” contained formant transitions from /s/, while the /i/ vowels from “she” contained formant transitions from /∫/, etc.

***Spectral degradation: noise-band vocoding***. For the NH listeners in simulated conditions, spectral resolution was degraded using noise-band vocoding (NBV), which is a common way to simulate a cochlear implant (see Shannon et al., 1995). This was accomplished using online signal processing within the iCAST stimulus delivery software (version 5.04.02; Fu, 2006), which is freely available at www.emilyshannonfufoundation.org. Stimuli were bandpass filtered into eight frequency bands using sixth-order Butterworth filters (24 dB/octave). This number of bands was chosen to best approximate the performance of CI users (Friesen et al., 2001). The temporal envelope in each band was extracted by half-wave rectification and low-pass filtering with a 300-Hz cutoff frequency, which was sufficient for temporal coding of the fundamental frequencies for all talkers used for these stimuli. The envelope of each band was used to modulate the corresponding bandpass-filtered noise. Specific band frequency cutoff values were determined assuming a 35 mm cochlear length (Greenwood, 1990) and are listed in Table 3. The lower and upper frequency cutoffs for the analysis and carrier bands were 150 and 10000 Hz, respectively, which is slightly expanded beyond the range commonly used in modern CI speech processors in order to capture a majority of the fricative spectrum. It is important to note that energy falling across channel boundaries is reflected as relative amplitude (envelope) differences for those channels, and overlap of energy in adjacent analysis bands is coded as temporal fluctuations reflecting the sum of the envelopes.

**Table 3:** **Analysis and carrier filter band corner frequencies for the noise vocoder in experiments 1 and 2**.

**Channel**	**1**	**2**	**3**	**4**	**5**	**6**	**7**	**8**
High-pass (Hz)	150	314	570	967	1586	2549	4046	6376
Low-pass (Hz)	314	570	967	1586	2549	4046	6376	10000

### Procedure

After hearing each stimulus, listeners used a computer mouse to select the word that they perceived in a four-alternative forced-choice task (the choices were “see,” “sue,” “she,” “shoe”). For NH listeners, stimuli were presented in ten alternating blocks of spectral resolution (unprocessed or 8-channel NBV), and presentation of tokens within each block was randomized. Each of the 144 stimuli was heard a total of 5 times in each condition of spectral resolution. CI listeners only heard the unprocessed stimuli. All testing was conducted in a double-walled sound-treated booth. Stimuli were presented at 65 dBA in the free field through a single loudspeaker.

### Analysis

Listeners' responses were analyzed using a generalized linear (logistic) mixed-effects model (GLMM) in the R software interface (R Development Core Team, 2010), using the lme4 package (Bates and Maechler, 2010). A random intercept (participant) effect was used, and the fixed-effects were the stimulus parameters described earlier (frication spectrum peaks, talker gender, vowel, formant transitions). The binomial family call function was used because responses were coded in a binary fashion as /∫/-onset or /s/-onset (i.e., 0 or 1 re /s/). Although the spectral peaks are represented on the figures using the Hz frequency scale, they were coded in the statistical model using centered log Hz (i.e., 0 for the median value).

Starting with an intercept-only model, factor selection (e.g., the inclusion of talker gender as a response predictor) was done using a forward-selection hill-climbing stepwise process whereby candidate factors were added one-by-one; the factor which yielded the lowest entropy was entered first. Subsequent factors (or factor interactions) were retained in the model if they significantly improved the model without over-fitting. The ranking metric (and test of significance for the inclusion of factors) was the Akaike information criterion (AIC) (Akaike, 1974), as it has become a popular method for model selection (Vaida and Blanchard, 2005; Fang, 2011). This criterion measures relative goodness of fit of competing models by balancing accuracy (likelihood) and complexity of the model. There were three sets of data: (1) NH listeners in the unprocessed sound condition, (2) NH listeners in the degraded condition, and (3) CI listeners in the unprocessed sound condition.

### Results and discussion

**Figure 1:**
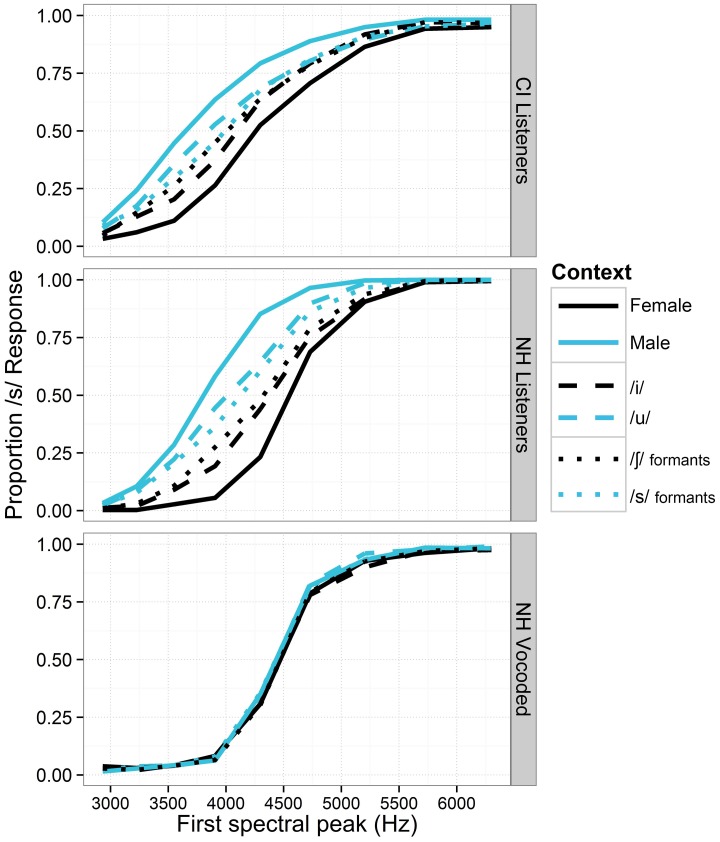
**Proportion of fricatives perceived as /s/ along the continuum of fricative spectra for all listener groups in Experiment 1.** For each cue level, responses are collapsed across all levels of the other context types (i.e., for the gender contrasts, data are collapsed across both /i/ and /u/ vowel contexts, and both levels of the formant transition cue). Black lines depict cue levels expected to produce fewer /s/ responses. Sensitivity to context is demonstrated by the space between same-shaped lines of different colors.

Identification functions for fricatives in the various vocalic contexts are shown in Figure 1. These data all replicate earlier findings of context effects for normal-hearing listeners; the effect of talker gender had the strongest effect on the identification functions, followed by vowel context and formant transitions. The generalized linear mixed-effects models revealed the effects shown in Table 4. For each model term in Table 4, we provide the Wald statistic (*z*-value), which tests the hypothesis that the effect of the factor is zero.

**Table 4:** **Main effects and interactions in experiment 1**.

	**NH unprocessed**	**NH vocoded**	**CI**
**MAIN EFFECTS**			
Fricative	22.86^***^	30.56^***^	24.05^***^
Gender	17.63^***^	n.s.	15.48^***^
Vowel	7.00^***^	n.s.	3.33^***^
Formant	8.18^***^	2.07^*^	n.s.
**INTERACTIONS**			
Fricative: gender	2.29^*^	N/A	n.s.
Fricative: vowel	n.s.	2.24^*^	3.29^*^
Fricative: formant	2.631^*^	n.s.	N/A
Gender: vowel	4.681^***^	N/A	n.s.
Gender: formant	n.s	N/A	N/A
Vowel: formant	n.s.	n.s	N/A

NH, Normal hearing; CI, Cochlear implant; N/A, main effect absent; no potential interaction. Numbers represent Wald statistics (z-values).

*** p < 0.001;

* p < 0.05.

For the NH listeners in the unprocessed sound condition, there were significant main effects of fricative spectrum, talker gender, vowel context, and formant transitions (all *p* < 0.001), in the expected directions (i.e., fricatives were more likely to be perceived as /s/ when in the context of male voices, /u/ vowels and formant transitions from /s/). There was a significant interaction between talker gender and vowel (the effect of gender was stronger for the /u/ vowel, *p* < 0.001). There were significant interactions between fricative spectrum and all three other main effects. The fricative spectrum cue was significantly weaker when the formant transition indicated /s/ (*p* < 0.01) and when the talker was male (*p* < 0.05). The fricative cue was marginally stronger when the vowel was /u/ (*p* = 0.055), and the inclusion of that interaction significantly strengthened the model. Wald statistics for each main effect suggested that the fricative cue was the strongest cue (*z* = 22.86), followed by talker gender, formant transitions, and vowel context (*z* = 17.63, 8.18 and 7.00, respectively).

For NH listeners in the vocoded condition, there were significant main effects for fricative spectrum (*p* < 0.001) and formant transitions (*p* < 0.05), and a marginally significant effect of vowel (*p* = 0.055), all in the expected directions. In general, the context effects in the degraded condition were negligible. Talker gender did not have a significant effect, despite yielding strongest context effect for unprocessed speech. Raw data for the degraded condition suggest that the effects of formant transition (*p* < 0.05) and vowel were present for only the median (most ambiguous) step of the fricative continuum. There was a significant interaction between fricative and vowel; the fricative effect was significantly larger when the vowel was /u/ (*p* < 0.05). All four cue factors (fricative, gender, vowel, and formant transitions) were significantly smaller in the vocoded condition compared to the unprocessed condition (all *p* < 0.001); interaction *z*-values of −5.55, −21.79, −10.20, and −5.12, respectively.

For the cochlear implant listeners, there were significant main effects of fricative spectrum, talker gender and vowel context (all *p* < 0.001), in the expected directions. The effect of formant transition facilitated perceptual bias in the correct direction but did not reach significance (*p* = 0.103), and its inclusion did not significantly improve the model. There was a significant interaction between fricative spectrum and vowel; the fricative effect was smaller when the vowel was /u/ (*p* < 0.001). The effect of the fricative cue was also significantly smaller when the talker was male (*p* < 0.05). There was no significant relationship between context effects and scores on consonant, vowel or sentence recognition (the correlation between vowel recognition and gender context effect approached significance; *t* = 2.32, adjusted *p* = 0.07).

Given the heterogeneity of the CI and NH groups in terms of age and hearing history, as well relatively small sample size in the CI group, direct statistical comparisons between groups should be treated with caution. A rough qualitative assessment suggests that CI listeners' vocalic context effects were smaller than those of NH listeners, but greater than those of NH listeners in the degraded condition, for whom there were virtually no context effects (see Figure 1). A GLMM using the data from NH listeners (non-vocoded signal only) and CI listeners did reveal significant interactions of hearing status with all four effects (fricative, gender, vowel and formant transitions), with interaction *z*-values of −17.19, −13.11, −9.55, and −5.86, respectively (all *p* < 0.001; all negative numbers indicate that the effects were smaller for the CI listener group).

**Figure 2:**
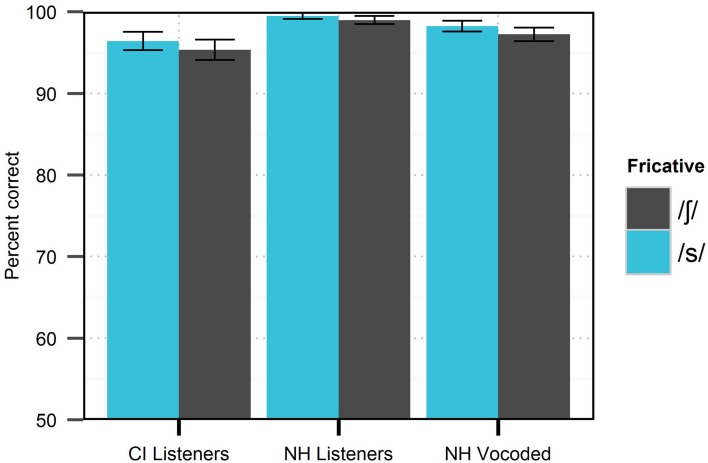
**Mean accuracy in identification of /s/ and /∫/ stimuli at fricative continuum endpoints by different listener groups in Experiment 1.** Error bars reflect standard error.

Context effects observed in this experiment cannot be inferred from performance on conventional word/phoneme recognition tests. Stimuli at the extreme endpoints of the fricative continuum are comparable to those that would be heard in such tests, and therefore, can be evaluated for correctness. Identification of such endpoint stimuli in this experiment was excellent for all listener groups (at least 95% for both phonemes, see Figure 2), despite marked differences in the use of context. This observation is consistent with the observation that differences in psychometric functions are primarily constrained to the center of the continuum, away from canonical clear pronunciations.

## Experiment 2: auditory and visual phonetic context effects

A second experiment was designed to test the influence of visual context cues on the perception of fricatives. This was a modified replication of Strand and Johnson (1996), with a number of important differences. The talkers used in this experiment were *not* chosen for their gender atypicality; on the contrary, the two talkers chosen for Experiment 2 were those who produced the most clearly differentiated responses in the audio-alone condition of Experiment 1. That is, they were the most stereotypically “male” or “female” voices, according to the fricative identification curves. Additionally, we tested listeners with CIs to examine whether visual cues would be utilized more heavily to accommodate gender-related speech differences. As in Experiment 1, we also tested NH individuals with spectrally degraded speech.

### Materials and methods

#### Participants

Participants included 10 adult (mean age 22.2 years; 6 female) listeners with NH [American National Standards Institute (ANSI), 2007]. Seven of these listeners also participated in Experiment 1. A second group of participants included seven adult CI users who previously participated in Experiment 1.

#### Stimuli

***Acoustic components***. The stimuli in Experiment 2 were a subset of those used in Experiment 1. The entire nine-step fricative continuum was used, but Experiment 2 used only two of the four talkers, and only the vowels with /s/ formant transitions (i.e., excised from words originally containing /s/; based on data from experiment 1, this was expected to yield a /s/ bias of roughly 1.3–5.2%, depending on the listener group). However, each of these four syllables is easily perceptible with either configuration of formant transitions and thus, the four alternative word choice was still a viable task. Both vowels /i/ and /u/ were used.

***Spectral degradation: noise-band vocoding***. Software used to deliver the first experiment was not suited for video presentation, so the second experiment was delivered using the Alvin software package (Hillenbrand and Gayvert, 2005); Alvin does not feature online vocoding, so vocoded stimuli were prepared offline using TigerCIS (Fu, 2010), a program with the same developer as the program used in Experiment 1. The vocoder analysis and carrier filter parameters were exactly the same as those used for Experiment 1.

**Figure 3:**
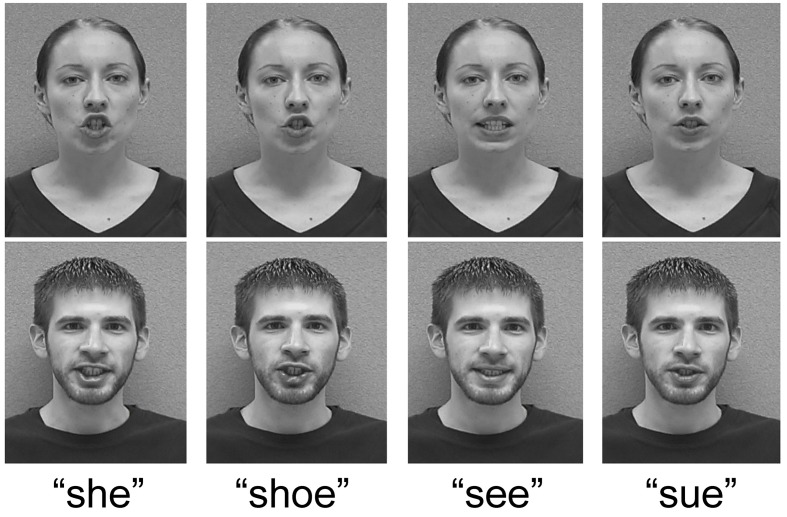
**Example frames from the videos used in Experiment 2, cropped to highlight the talkers' faces.** Images were taken from portions of videos during consonant articulation. Lip rounding coarticulation is visible for all consonants in the context of the /u/ vowel.

***Video stimulus recording***. Two speakers (one male and one female native English speaker; the first and second author) were recorded in front of a gray background. There were eight video tokens (one of each speaker saying the words “see,” “she,” “sue,” and “shoe”) to be combined with the auditory stimuli from Experiment 1. Video and audio were recorded concurrently with a Canon DM-XL1 video camera onto digital videotape (mini DV; 29.97 frames per second) and imported as audiovisual interleave (AVI) files for offline segmentation. Audio obtained from the video recording was used to determine fricative onset and overall token duration for alignment with audio stimuli from Experiment 1. Dubbed AVI files were converted to grayscale to reduce visual complexity, cropped to include only the talkers' upper shoulders and face (final dimensions: 425×425 pixels), and compressed with Cinepak in VirtualDub (www.virtualdub.org) to reduce file size. See Figure 3 for sample frames from the videos used in Experiment 2.

***Audio-visual crossings***. All factors were crossed with the exception of vowel audio and vowel video, which were always consistent. Crossed factors included: 2 faces (female, male) × 2 voices (female, male) × 9 fricative continuum tokens × 2 visual lip configurations (rounded or unrounded for the consonant) × 2 vowels (/i/ and /u/, with accompanying lip posture). This resulted in a total of 144 dubbed audio-visual tokens for each condition (unprocessed/normal and spectrally-degraded).

#### Procedure

The procedure for Experiment 2 was nearly the same as for Experiment 1, with the exception of the software used to deliver the stimuli. Video stimuli were centered on the display screen, with the four word choices equally spaced around the periphery. Responses were visible during the videos, which all began and ended with neutral closed lip posture.

#### Analysis

Listeners' responses were fit using the same GLMM procedure as for Experiment 1, with additional fixed-effects (fricative spectrum peaks, audio gender, video gender, vowel, consonant lip rounding, coded by whether the original video recording was from a /s/-onset or /∫/ word).

#### Results and discussion

**Figure 4:**
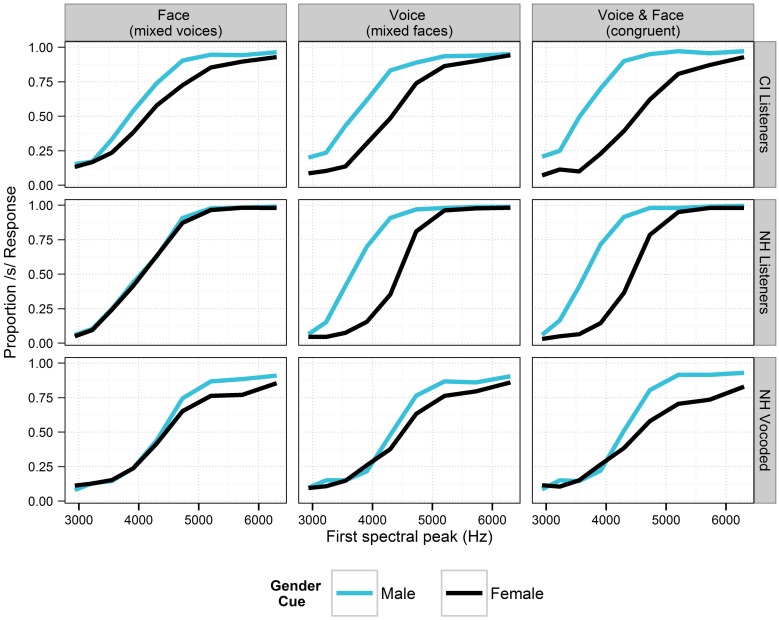
**Perception of the fricative continuum in the context of various auditory, visual and audio-visual cues to talker gender for listeners in Experiment 2.** For each cue, responses are collapsed across all levels of the other cues (i.e., the data include all levels of vowel context and consonant lip rounding). The “Face” and “Voice” columns show data where the opposite cue is free to vary (i.e., a female face with either a female or a male voice). Stimuli where the voice and face cues were of the same gender are illustrated in the rightmost panels. Black lines depict cue levels expected to produce fewer /s/ responses. Sensitivity to context is demonstrated in each panel by the space between lines of different colors.

**Figure 5:**
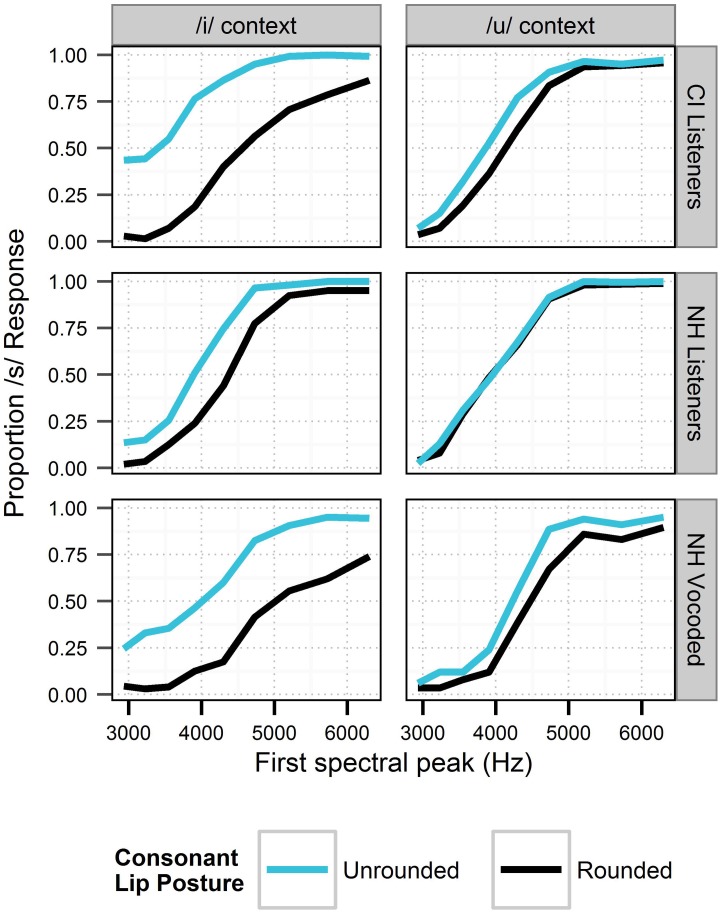
**Perception of the fricative continuum in the context of visual cues to consonant lip rounding and vowel context in Experiment 2.** For each cue, responses are collapsed across all levels of the gender cues. Black lines depict cue levels expected to produce fewer /s/ responses. Sensitivity to context is demonstrated in each panel by the space between lines of different colors.

Identification functions in the various auditory and visual contexts are shown in Figures 4, 5. The generalized linear mixed-effects models revealed the effects shown in Table 5. For each model term in Table 5, we provide the Wald statistic (*z*-value), which tests the hypothesis that the effect of the factor is zero.

**Table 5:** **Main effects and interactions in experiment 2**.

	**NH**	**NH**	**CI**
	**unprocessed**	**vocoded**	
**MAIN EFFECTS**			
Fricative	31.08^***^	17.47^***^	20.32^***^
Gender–Voice	13.43****	5.85^***^	9.70^***^
Gender–Face	n.s.	7.52^***^	10.14^***^
Vowel	3.31^***^	8.28^***^	7.4^***^
Lip rounding	13.52^***^	20.85^***^	18.84^***^
**INTERACTIONS**			
Fricative: gender (voice)	n.s.	3.56^***^	2.38^*^
Fricative: gender (face)	n.s.	3.83^***^	2.15^*^
Fricative: vowel	7.05^***^	8.77^***^	4.28^***^
Fricative: lip rounding	n.s.	n.s.	n.s.
Gender (voice): gender (face)	N/A	n.s.	n.s.
Gender (voice): vowel	10.55^***^	n.s.	n.s.
Gender (voice): lip rounding	n.s.	n.s.	n.s.
Gender (face): vowel	N/A	3.28^**^	4.20^***^
Gender (face): lip rounding	N/A	5.55^***^	3.07^***^
Vowel: lip rounding	8.14^***^	9.68^***^	11.95^***^

NH, Normal hearing; CI, Cochlear implant; N/A, main effect absent; no potential interaction. Numbers represent Wald statistics (z-values), which reflect the strength of each factor in the model.

*** p < 0.001;

** p < 0.01;

* p < 0.05.

For the listeners with NH in the unprocessed (non-vocoded) condition, there were significant main effects of fricative spectrum, lip rounding, vowel environment, and auditory gender (voice) cues (all *p* < 0.001), in the expected directions (i.e., fricatives were more likely to be perceived as /s/ when in the context of male voices, /u/ vowels and unrounded lips). The effect of visual gender cues did not reach significance (*p* = 0.078). The effect of consonant lip rounding was significantly weaker for words with /u/ (*p* < 0.001), likely because anticipatory lip rounding is customary in that vowel environment (Daniloff and Moll, 1968), and thus, would not necessarily be a cue for the consonant. The fricative spectrum effect was significantly larger in the context of /u/ (*p* < 0.001), consistent with the increased load of the fricative when lip-rounding is rendered predictable by the vowel environment. The effect of vowel context was larger for the male talker (*p* < 0.001), likely because the female talker exhibited more /u/-fronting (a more anterior tongue position for /u/, thus, reducing its acoustic distinctiveness from /i/ in the domain of the second formant). For listeners with NH, the sizes of the fricative effect and auditory gender cue effect in experiment 2 were both relatively smaller than those found in experiment 1, where there were no visual cues.

For NH listeners in the vocoded condition, each main effect reached significance (all *p* < 0.001). The consonant lip rounding cue was significantly weaker for words with /u/ (*p* < 0.001), and was stronger for the female face (*p* < 0.001). The effect of vowel was weaker for the male face (*p* = 0.001). The fricative spectrum cue was stronger for the male voice, the male face, and in the context of /u/ (all *p* < 0.001). The effects of fricative and gender were significantly smaller for NH listeners when the signals were vocoded (both *p* < 0.001, *z*-values of −12.79 and −7.73, respectively), while there significant increases in the effects of lip rounding (*p* < 0.001; *z* = 4.61) and face (*p* < 0.01; *z* = 2.76). The size of the vowel context effect did not significantly differ across listening conditions. For listeners with NH in the degraded condition, the size of the fricative effect in experiment 2 was relatively smaller than that found in experiment 1, where there were no visual cues. The size of the auditory gender cue effect was larger in experiment 2; perhaps the inclusion of both auditory and visual cues highlighted gender as a potential cue in the second experiment.

For listeners with CIs, each main effect reached significance (all *p* < 0.001). The relative strength of the two cues to gender was affected by vowel environment. In the context of /u/, the auditory cues (fricative spectrum) were dominant, while in the context of /i/, the visual cues (lip rounding) were dominant. The effect of lip rounding was larger for the female face (*p* < 0.01) and weaker in the context of /u/ (*p* < 0.001). The effect of vocal gender cues was larger in the context of /u/. The fricative spectrum cue was stronger for the female voice (*p* < 0.05), and stronger for the male face (*p* < 0.05). Additionally, the fricative spectrum effect was considerably larger in the context of the /u/ vowel (*p* < 0.001), consistent with the increased load of the fricative when lip-rounding is rendered predictable by the vowel environment (to be discussed further below). The size of the fricative effect was marginally larger when lips were unrounded (*p* = 0.075), but the inclusion of that interaction did not significantly improve the model. For listeners with CIs, the sizes of the fricative effect and auditory gender cue effect in experiment 2 were both relatively smaller than those found in experiment 1, where there were no visual cues. There were no significant correlations between context effects in experiment 2 and any of the speech recognition scores.

As for experiment 1, the statistical comparison between NH and CI listener groups in the second experiment should be interpreted with caution, given the sizes and nature of these groups. There was a significant interaction between hearing status and each of the five main effects. For CI listeners, there were significantly smaller effects of the fricative cue (*p* < 0.001; *z* = −9.162) and voice cues to gender (*p* < 0.05; *z* = −2.512). On the other hand, listeners with CIs made significantly greater use of cues to lip-rounding (*p* < 0.001; *z* = 6.450), vowel context (*p* < 0.05; *z* = 2.066), and face cues to gender (*p* < 0.001; *z* = 4.564).

## Summary of results

In Experiment 2, listeners were presented with a subset of the sounds from Experiment 1, with accompanying visual cues. The auditory gender context effects from Experiment 1 were replicated, with additional effects of visual cues to gender (face) and lip rounding. Results suggest that although CI listeners receive degraded auditory cues to gender (Fu et al., 2005), they showed sensitivity to auditory cues to gender when identifying fricatives in Experiment 1. Additionally, CI listeners in experiment 2 were able to utilize visual cues to complement auditory cues to achieve a level of contextual sensitivity comparable to that observed in NH listeners. The simultaneous visual presentation of a female or male face affected /s/ and /∫/ categorization considerably for listeners with CIs, presumably because these listeners generally rely more heavily on non-auditory sources of information. The accommodation of gender context using visual cues was modest for NH listeners in the spectrally-degraded condition, where context effects were fairly small overall.

Listeners (especially those with CIs) demonstrated nuanced sensitivity to lip posture, reflecting tacit understanding of visual cues for the consonant and vowel as a coherent unit rather than as individual segments. Given an interpretation of the vowel as /i/, lip rounding had to be attributed to the consonant. Thus, when in the context of /i/, consonant lip rounding gave rise to considerable bias toward /∫/ even at high-frequency steps of the fricative continuum; the fricative itself was a weaker cue in this case. Conversely, in the context of the rounded vowel /u/, lip rounding during the consonant is acceptable for both /s/ and /∫/. Accordingly, in the context of /u/, the CI listeners were relatively less affected by lip rounding and relatively more affected by the spectrum of the fricative. This pattern of second-order context effects (use of the lip-rounding context according to the level of the vowel context) is illustrated by the psychometric functions in Figure 5, where lip-rounding-driven differences in psychometric functions are greater in the left (/i/) panels than in the right (/u/) panels. The influence of lip posture on the perception of speech by listeners with CIs is not surprising in the context of previous literature, but it is noteworthy that perception corresponded not only with the target segments, but the segments in the context of the entire syllable.

## Discussion

The presence of auditory phonetic context effects in this study supported findings of earlier literature and generalized the phenomenon to CI listeners. Specifically, listeners with NH were more likely to identify fricatives as /s/ when they were (1) perceived to be spoken by male voices, (2) in the context of a rounded vowel, or (3) followed by formant transitions appropriate for /s/. CI listeners showed auditory context effects of a similar type but arguably to a lesser degree. The differences between alternate contexts in this experiment (male/female voices, /i/-/u/ vowels, /s/-/∫/ formants) are all cued primarily by spectral properties such as formant spacing, spectral tilt, voice pitch, vowel formants, and dynamic formant transitions; the presence of context effects for CI listeners was somewhat surprising, given the limitations of spectral resolution in CIs. To the extent that these effects are representative of the many inter-talker and cross-context variations present in natural speech, successful accommodation appears to emerge without fine spectral resolution.

The 50% crossover point in /s/-/∫/ identification in each context was calculated from the group aggregate GLMMs in both experiments. In Table 6, we convert the effect of context into its equivalent shift in fricative spectrum peak frequency, as a kind of “common currency” for context effects. For example, changing the gender of the talker from female to male in experiment 1 had an effect equivalent to raising the fricative spectrum peak by roughly 700 Hz for listeners with NH.

**Table 6:** **Difference (in Hz) between /∫/- /s/ category boundaries at opposing levels of each context tested in both experiments (modeled from GLMM results)**.

	**NH listeners**	**NH vocoded**	**CI listeners**
**EXPERIMENT 1 (AUDIO ONLY)**			
Gender (voice)	702	0	636
Vowel	282	62	168
Formant	242	51	0
**EXPERIMENT 2 (AUDIO-VISUAL)**			
Voice	675	183	490
Face	0	176	290
Voice and face	675	358	780
Lips	274	1016	993
Vowel	131	247	98

Calculation: the NH listener category boundary in Experiment 1 for female voices was 4553 Hz and for male voices was 3851 Hz; the difference is 702 Hz. A difference of 0 means that the effect was not significant in the statistical model and therefore, yielded no predicted effect of context.

Time-varying spectral contrast encapsulates some of the acoustic variables that underlie the contextual accommodation in this study and other previous studies (Lotto and Kluender, 1998). For example, the perception of /s/ can be described as perception of high contrast (relative to that found in /∫/) between the fricative and the adjacent vowel in the region of F3 and F4 (consistent with the framework used by Hedrick and Carney, 1997). Given the same fricative sound, the upward shift in formant frequencies (toward the frequencies with frication energy) for the female voice would result in less contrast in this region, necessitating a higher-frequency spectral peak in order to be perceived as /s/. Framing the fricative contrast in terms of general auditory contrast is consistent with the report by Bladon et al. (1984) that suggested that vocal tract differences (and hence, *perceptions* of vocal tract differences) are not sufficient to drive the gender-related differences in speech production/perception. Contrast sensitivity appears to be a domain-general principle of sensory systems (Kluender et al., 2003), and has been shown to explain a significant proportion of speech intelligibility (Stilp and Kluender, 2010). Recent efforts by Alexander et al. (2011) show that enhancement of this type of contrast can lead to improved performance on speech recognition, particularly for speech contrasts that are notoriously difficult for CI listeners (e.g., place of articulation). The observation that the contrasts in the current study were perceptible by CI listeners shows promise for coding strategies that capitalize on time-varying spectral change.

It is commonplace in CI research to use vocoders to produce “simulated CI” speech that is then played for NH listeners. Results presented in this study expose some limitations of the conventional vocoder method in modeling perception of speech by CI listeners. Although performance for clearly-pronounced words was comparable across all groups of listeners (Figure 2), CI listeners showed markedly greater accommodation to phonetic contexts than NH listeners in the degraded condition (Table 6). Experience/familiarity with signal degradation may partly explain the disparity in results. While listeners with NH were exposed to a novel type of speech signal, listeners with CIs simply heard speech as they typically would, with their everyday processor settings. Despite the difference in experience between the listener groups, it is notable that NH listeners in the degraded condition showed virtually no auditory accommodation in Experiment 1 (Figure 1), as several published reports suggest that performance on speech recognition by NH listeners in vocoded conditions is typically *better* than that of high-performing CI listeners (c.f. Friesen et al., 2001).

In Experiment 2, people with CIs utilized visual talker information to accommodate gender-driven acoustic differences in speech production, perhaps as a supplement to or proxy for degraded auditory information. There is precedent for increased cross-modal influence on speech perception, especially in the presence of acoustic signal degradation (e.g., Sumby and Pollack, 1954). Compared to listeners with NH, listeners with hearing impairment are more heavily influenced by visual cues like lip posture, especially when perceiving consonant place of articulation (Walden et al., 1990, 2001). It is customary for CI users to be trained (or at least encouraged) to use visible speech cues to facilitate comprehension of spoken speech (Lachs et al., 2001; Strelnikov et al., 2009; Barone et al., 2010) in addition to auditory training techniques. Through a number of evaluation measures, it has been shown that speechreading ability in both hearing-impaired and NH populations is highly variable (Bernstein et al., 1998; Woodhouse et al., 2009). However, these studies generally suggest that relative to auditory-alone conditions, intelligibility of audio-visual speech is nearly always improved (Grant et al., 1998).

It is noteworthy that the effect in this experiment went in the direction predicted by the acoustics that typically correspond to the gender of the visual stimulus; listeners had no reason to prefer /s/ when seeing male faces (especially when presented with concurrent female voices) other than having been exposed to the natural association between visual cues and the auditory spectral properties of voices that correspond to those faces. The influence of this cue cannot be completely understood on the basis of this experiment alone, but it is likely that it arises from a learned association between phonetic segments and typical gender-driven differences in speech production. Although Holt et al. (2005) suggest that mere variation in concurrent visual information is sufficient to induce context effects, there was no co-varying acoustic (spectral) cue in the current experiment because the visual and auditory cues were orthogonal.

There were two distinct effects of visual speech information in this study. First, lip rounding during the consonant segment increased the proportion of /∫/ responses in a predictable fashion. The effect of lip rounding was further modulated by vowel context, suggesting that listeners are sensitive to the compatibility of auditory and visual phonetic cues at the syllabic level in addition to the segmental level. This was especially apparent for the NH listeners in spectrally-degraded conditions and CI listeners (Figure 5). Listeners' responses suggest sensitivity to lip rounding cues was consistent with compatibility of lip rounding with each of the consonant and vowel combinations. The integration of auditory and visual phonetic cues appears to operate over a time window that includes both segments.

The use of context and visual cues has potential impact on the rehabilitation of listeners with CIs. Consonant and vowel recognition performed in predictable syllable contexts (e.g., /apa/, /ata/, /aka/, etc.) is not reflective of the variability in real speech signals (both linguistically and acoustically), which is an important part of basic speech recognition in everyday life. It is not enough for listeners to recognize cues for a consonant in just one environment; they must be able to accommodate variability arising from different phonetic contexts and from different talkers; basic word identification is not sensitive enough to capture this ability (Figure 2). It is likely that the loss of the rich redundancy in continuous speech might hinder intelligibility by CI listeners, forcing them to rely more heavily on linguistic context and expend more cognitive resources. Given the rapid rate of speech, the amount of acoustic contextual acoustic information that is potentially lost is considerable. Although CI listeners exceeded our expectations in this study, the tasks presented here may represent only a fraction of the challenges faced in everyday conversation.

Finally, visual cues clearly provide information that can be of use to listeners with hearing impairment. To the extent that audiologists aim to equip clients with all the tools necessary to succeed in everyday listening, it may be beneficial to exploit the relationships between visual and auditory cues to facilitate not only consonant recognition (Bernstein et al., 1998), but also accommodation to inter-talker speech variability. The shift of focus from sentence recognition to word recognition to phonetic cue-weighting to inter-talker accommodation represents different levels of granularity in speech perception, which is highly desirable when evaluating listeners with hearing impairment. Given the highly variable nature of speech acoustics across talkers, testing fine-grained abilities such as cue-weighting and context accommodation may reveal important limiting factors in performance, and may serve as tools for new exploration as technology continues to improve.

### Conflict of interest statement

The authors declare that the research was conducted in the absence of any commercial or financial relationships that could be construed as a potential conflict of interest.

## Footnotes

^1^A Praat script that can be used to re-create this continuum is available upon request from the first author.
